# Nitric oxide in guard cells as an important secondary messenger during stomatal closure

**DOI:** 10.3389/fpls.2013.00425

**Published:** 2013-10-29

**Authors:** Gunja Gayatri, Srinivas Agurla, Agepati S. Raghavendra

**Affiliations:** Department of Plant Sciences, School of Life Sciences, University of HyderabadHyderabad, India

**Keywords:** abscisic acid, cytosolic pH, elicitors, polyamines, phospholipids, reactive oxygen species, signal transduction

## Abstract

The modulation of guard cell function is the basis of stomatal closure, essential for optimizing water use and CO_2_ uptake by leaves. Nitric oxide (NO) in guard cells plays a very important role as a secondary messenger during stomatal closure induced by effectors, including hormones. For example, exposure to abscisic acid (ABA) triggers a marked increase in NO of guard cells, well before stomatal closure. In guard cells of multiple species, like *Arabidopsis*, *Vicia* and pea, exposure to ABA or methyl jasmonate or even microbial elicitors (e.g., chitosan) induces production of NO as well as reactive oxygen species (ROS). The role of NO in stomatal closure has been confirmed by using NO donors (e.g., SNP) and NO scavengers (like cPTIO) and inhibitors of NOS (L-NAME) or NR (tungstate). Two enzymes: a L-NAME-sensitive, nitric oxide synthase (NOS)-like enzyme and a tungstate-sensitive nitrate reductase (NR), can mediate ABA-induced NO rise in guard cells. However, the existence of true NOS in plant tissues and its role in guard cell NO-production are still a matter of intense debate. Guard cell signal transduction leading to stomatal closure involves the participation of several components, besides NO, such as cytosolic pH, ROS, free Ca^2+^, and phospholipids. Use of fluorescent dyes has revealed that the rise in NO of guard cells occurs after the increase in cytoplasmic pH and ROS. The rise in NO causes an elevation in cytosolic free Ca^2+^ and promotes the efflux of cations as well as anions from guard cells. Stomatal guard cells have become a model system to study the signaling cascade mechanisms in plants, particularly with NO as a dominant component. The interrelationships and interactions of NO with cytosolic pH, ROS, and free Ca^2+^ are quite complex and need further detailed examination. While assessing critically the available literature, the present review projects possible areas of further work related to NO-action in stomatal guard cells.

## Introduction

Stomatal pores are the gateways for not only transpirational H_2_O loss but also entry of CO_2_ into leaves. Due to such dual role, the regulation of stomatal aperture, and yet maintenance of opening are essential to keep up the water balance and at the same time make CO_2_ available for photosynthesis. Stomatal opening and closure are mediated by the changes in turgor pressure of guard cells. Stomata open when guard cells are turgid and close when the guard cells are flaccid. As closed stomata restrict pathogen entry into leaves, stomata become key players also in defense response against several pathogens (Underwood et al., 2007; Melotto et al., 2008). Several factors modulate stomatal function, such as drought, light, high CO_2_, humidity, and plant hormones, such as ABA (all abbreviations listed on first page). Some of the plant hormones (ABA, MJ, ethylene), salicylic acid, polyamines and even elicitors (mostly microbial) cause stomatal closure, while auxins and cytokinins promote stomatal opening (Bright et al., 2006; Acharya and Assmann, 2009; Alcázar et al., 2010; Jing et al., 2012; Ye et al., 2013).

NO has multifunctional roles in plants: stomatal movement, host-pathogen interactions, hormonal signaling during growth/development and adaptation to abiotic/biotic stress (Delledonne et al., 1998; Bright et al., 2006; Yan et al., 2007; Neill et al., 2008; Wilson et al., 2008, 2009; Siddiqui et al., 2011). In plants, NO can be a signal to induce secondary metabolite accumulation (Lu et al., 2011) and to promote cell death (Gupta et al., 2011b Bellin et al., 2013) The production of NO in stomatal guard cells has been known since several years (Desikan et al., 2002; Garcia-Mata et al., 2003). But the mechanisms of NO action and interaction with other signaling components in guard cells have been studied in detail, since only a few years. The rise in NO of guard cells is a common and dominant event during stomatal closure induced by several effectors and in different plants (Table 1).

**Table 1:** **The rise in NO of guard cells as a common event during stomatal closure induced by hormones, elicitors or environmental factors**.

**Effector**	**Source *in vivo***	**Test plant**	**References**
**PLANT HORMONES**			
ABA	Endogenous	*Vicia faba*	García-Mata and Lamattina, 2002
		*Pisum sativum*	Gonugunta et al., 2008
		*Arabidopsis thaliana*	Neill et al., 2008; Islam et al., 2010
MJ	Endogenous	*A. thaliana*	Munemasa et al., 2007; Saito et al., 2009
		*V. faba*	Xin et al., 2005
SA	Endogenous	*V. faba, Commelina communis*	Xin et al., 2003
		*A. thaliana*	Sun et al., 2010; Khokon et al., 2011
		*Lycopersicon esculentum*	Poór and Tari, 2012
Ethylene	Endogenous	*A. thaliana*	Jing et al., 2010
		*V. faba*	Liu et al., 2012
**BIOTIC STRESS COMPONENTS (ELICITORS)**			
Chitosan	Derivative of chitin fragments from fungal cell wall	*L. esculentum, C. communis*	Lee et al., 1999
		*P. sativum*	Srivastava et al., 2009
		*A. thaliana*	Khokon et al., 2010b
Flg22^*^	22 amino acid peptide from Flagellin, bacterial flagellar protein	*A. thaliana*	Melotto et al., 2006
LPS^*^	Glycolipid component of gram negative bacterial outer membrane	*A. thaliana*	Melotto et al., 2006
*E. coli* O157:H7	Human pathogen	*A. thaliana*	Melotto et al., 2006
Harpin	*Xanthomonas oryzae*	*Nicotiana benthamiana*	Zhang et al., 2009a, 2012b
INF1	*Phytophthora infestans*	*N. benthamiana*	Zhang et al., 2009a
Boehmerin	*Phytophthora boehmeriae*	*N. benthamiana*	Zhang et al., 2009a, 2012b
Nep1	*Magnaporthe oryzae*	*N. benthamiana*	Zhang et al., 2012b
YEL (Yeast elicitor)	Yeast extract	*A. thaliana*	Khokon et al., 2010a
Oligochitosan	Fragment of chitosan prepared by enzymatic hydrolysis	*Brassica napus*	Li et al., 2009b
**ENVIRONMENTAL FACTORS**			
UV-B	Environment	*V. faba*	He et al., 2005
		*A. thaliana*	He et al., 2013
Bicarbonate (mimics high CO_2_)	Environment	*P. sativum*	Kolla and Raghavendra, 2007
**SIGNALING COMPONENTS**			
CaCl_2_ (Buffered)	Endogenous	*A. thaliana*	Wang et al., 2012
H_2_O_2_	Endogenous	*V. faba*	He et al., 2005
		*A. thaliana*	Bright et al., 2006
Calmodulin	Endogenous	*A. thaliana*	Li et al., 2009a

* PAMP- the term used for elicitors like flg22, LPS.

There has been growing interest in NO as an essential signal molecule during stomatal closure, and plant growth/development, besides defense against pathogens. The ABA-induced stomatal closure is associated with a rise in NO as well as ROS of guard cells. The rise in NO causes elevation of free Ca^2+^ in guard cells, restriction of K^+^ influx and promotion of anion efflux (Garcia-Mata et al., 2003; Sokolovski and Blatt, 2004), all resulting in loss of guard cell turgor and stomatal closure. This article emphasizes that NO is a common factor during stomatal closure induced by varying factors, including hormones, microbial elicitors (yeast/bacterial/fungal/pathogen) and abiotic environmental stresses. The possible sources of NO are described, highlighting the ambiguity on the role of true NOS in plants. A pathway of signal transduction, with the components involved in NO action, is proposed. Attention is drawn toward the interaction of NO with other signaling components in guard cells. Finally, a few of the emerging topics and unresolved questions, for further research are indicated.

In view of the large number of reports on the rise in NO of guard cells in relation to stomatal closure, we had to limit references to original articles, published in the last 5 years. There are excellent reviews covering the earlier work on the role of NO during stomatal closure (García-Mata and Lamattina, 2002, 2013; Neill et al., 2003, 2008; Desikan et al., 2004; Lamotte et al., 2005; Wilson et al., 2008, 2009; Hancock et al., 2011) and the importance of NO during the innate immunity responses of plants (Wendehenne et al., 2004; Leitner et al., 2009; Gaupels et al., 2011; Yoshioka et al., 2011). The importance of NO as a general signaling molecule in several processes of growth and development have been reviewed elsewhere (Durner and Klessig, 1999; Lamattina et al., 2003; Moreau et al., 2010; Baudouin, 2011; Fröhlich and Durner, 2011; Martínez-Ruiz et al., 2011; Astier et al., 2012; Simontacchi et al., 2013).

## Hormones: ABA, ethylene, methyl jasmonate

The rise in NO is a common step during stomatal closure induced by hormones like ABA; or elicitors like chitosan; and even abiotic stress conditions (Table 1). Among the plant hormones, the perception and action of ABA is well characterized (Sirichandra et al., 2009; Cutler et al., 2010; Raghavendra et al., 2010). The stomatal closure induced by ABA involves a series of events, including a rise in reactive nitrogen species i.e., nitric oxide (NO). Additional signaling components that are involved are: reactive oxygen species (ROS, mostly H_2_O_2_), cytosolic Ca^2+^, cytoplasmic pH, G-proteins, protein kinases as CDPK and MAPK, protein phosphatases, phospholipases and sphingolipids (Gonugunta et al., 2008; Neill et al., 2008; Wang and Song, 2008; Umezawa et al., 2010; García-Mata and Lamattina, 2013). Extensive studies on guard cells of *Arabidopsis*, pea, *Vicia faba* and *Commelina communis* have established that NO is an essential signaling component during ABA-induced stomatal closure (Xin et al., 2005; Gonugunta et al., 2008, 2009; Neill et al., 2008). The increase in NO is usually associated with the elevated ROS levels, particularly H_2_O_2_, generated by plasma membrane NADPH oxidase. The role of several signaling components involved in NO production and stomatal closure induced by ABA was convincingly demonstrated by studies performed in Arabidopsis mutants (Table 2). The impaired NO production by ABA in *nia1,nia2* mutants (Desikan et al., 2006) and in *atrbohD/F* mutant is an indication of the key roles of NR and NADPH oxidase, respectively (Bright et al., 2006).

**Table 2:** **Use of Arabidopsis mutants to demonstrate the importance of signaling components involved in the rise of NO during stomatal closure**.

**Mutant**	**Deficiency in mutant**	**Effector used for NO rise**	**Impairment in the plant**	**References**
*abi1-1* and *abi2-1*	Protein phosphatase 2C	ABA	Stomatal closure but not NO production	Desikan et al., 2002
*aba2-2*	Protein phosphatase 2C	Methyl jasmonate	NO and ROS production	Ye et al., 2013
*atrbohD/F*	NADPH Oxidase	ABA	H_2_O_2_ production	Bright et al., 2006
*coi1* and *abi2-1*	Coronatine-insensitive1 protein (COI1) and protein phosphatase 2C	Methyl jasmonate	ROS and NO production	Munemasa et al., 2007
*cpk6-1*	Calcium dependent protein kinase	ABA and MJ	NO levels; no change in ROS	Munemasa et al., 2011a
*gpa1-1, gpa1-2 atnoa1* and *atrbohD/F*	G-protein α sub unit and NADPH Oxidase	Extracellular calmodulin (ExtCaM)	NO rise in guard cell and stomatal closure	Li et al., 2009a
*nia1* and *nia2*	Nitrate reductase	Salicylic acid and ABA	NO rise in guard cell and stomatal closure	Bright et al., 2006; Hao et al., 2010
*pldα1*	Phospholipase Dα1	ABA	NO production	Zhang et al., 2009b
*Pldδ-1/pldα1*	Phosholipase Dα and Dδ	ABA	NO production only, but not stomatal closure	Distéfano et al., 2012
*rcn1*	Regulatory subunit of protein phosphatase 2A	Methyl jasmonate	NO production	Saito et al., 2009

The other hormones, which induce an increase in NO leading to stomatal closure, are ethylene and MJ. External application of ethephon (an ethylene-releasing compound) or 1-aminocyclopropane-1-carboxylic acid (the precursor of ethylene) induced stomatal closure in a dose-dependent manner in *Arabidopsis thaliana* (Desikan et al., 2006). Ethylene-induced stomatal closure was associated with a rise in not only NO, but also H_2_O_2_, Ca^2+^, and cytoplasmic pH (Jing et al., 2010, 2012). The precise order of these molecules during NO action and stomatal closure is not yet known. The effects of ethylene on NO level may be either direct or indirect through the modulation of endogenous ABA levels. This aspect needs additional experiments for confirmation.

MJ, a linolenic acid derivative, is as powerful as ABA in inducing stomatal closure, and elevating the levels of NO, besides ROS in guard cells (Gonugunta et al., 2009; Munemasa et al., 2011b). The role of NO as one of the signaling components during MJ-induced stomatal closure is further confirmed by the decrease in NO production and stomatal closure by L-NAME in *V. faba* guard cells (Xin et al., 2005). The MJ or ABA-induced NO production was impaired in *rcn1* mutant of *A. thaliana*, deficient in the regulatory subunit of protein phosphatase 2A (RCN1) (Saito et al., 2008, 2009). However, SNP (a NO donor) induced stomatal closure along with rise in guard cell NO levels in *rcn1* mutant as well as in wild type.

## Microbial elicitors

Besides being gateways for water/CO_2_, stomata can limit the invasion of pathogenic bacteria, and thus be a part of the plant innate immune system (Baker et al., 2010; Zeng et al., 2010). A burst in NO production has long been identified as one of the plant defense responses. Further, NO plays a very important role in cell death and activation of defense genes against plant pathogens (Delledonne et al., 2003; Romero-Puertas et al., 2004; Garcia-Brugger et al., 2006). The protective role of NO doubles up, as it upregulates secondary metabolism, and levels of antimicrobial compounds (Wang and Wu, 2004; Zhang et al., 2012a). In view of such crucial role, the molecular events in plant cells, triggered by NO, to help in innate immunity have been studied in detail. Compared to the extensive literature on the role of the NO-burst as a component of pathogen resistance, there is very limited work on the mechanism of NO-rise in guard cells, when exposed to elicitors/plant pathogens.

A typical effect of several elicitors is the marked stomatal closure and an increase in guard cell NO (Table 1). NO production was observed in guard cells of *A. thaliana*, *Pisum sativum*, and *Nicotiana benthamiana* in response to elicitors such as, PAMP, chitosan and oligochitosan (Melotto et al., 2006; Li et al., 2009b; Srivastava et al., 2009). In addition, other elicitors such as harpin, boehmerin, INF1, and Nep1 induced the production of NO in guard cells of *N. benthamiana* (Zhang et al., 2009a, 2012b). Impaired stomatal closure in response to elicitors by cPTIO (NO scavenger) or upon treatment with L-NNA (NOS inhibitor) confirms the role of NO in stomatal signaling (Melotto et al., 2006; Khokon et al., 2010a,b; Zhang et al., 2012b).

The production of NO occurred downstream of ROS, during stomatal closure induced by chitosan (Srivastava et al., 2009; Khokon et al., 2010b). The signaling components identified with elicitor-induced stomatal closure and NO-rise in guard cells are: ROS/NADPH oxidases, G-proteins, vacuolar processing enzyme (Zhang et al., 2009a, 2010, 2012b). It is not clear if the signal transduction chain involving NO-rise and stomatal closure induced by different elicitors follows the same or a modified pathway.

## Salicylic acid

SA is a phenolic compound, known to play a key role in a wide range of physiological and developmental processes, such as thermogenesis, fruit ripening, ethylene synthesis and plant defense against pathogens (Loake and Grant, 2007). There have been early reports on the regulation by SA of stomatal movement (Manthe et al., 1992; Lee and Joon-Sang, 1998) and role of signaling molecules, such as superoxide radicals, Ca^2+^, H_2_O_2_, and NO in modulating SA-effects (Mori et al., 2001). The SA-induced NO production and stomatal closure was impaired by cPTIO (NO scavenger) in guard cells of *V. faba* (Xin et al., 2003) and Arabidopsis (Khokon et al., 2011) highlighting the importance of NO during responses to SA.

## Phospholipids

Phospholipids are major components of plasma membrane and have emerged as key signaling molecules (Meijer and Munnik, 2003; Testerink and Munnik, 2005; Wang, 2005). These phospholipids such as phosphatidic acid (PA), phosphatidylinositol 4,5-bisphosphate (PIP_2_) and diacylglycerol (DAG) regulate a wide range of growth and developmental processes including ABA signaling, programmed cell death and defense response (Katagiri et al., 2005; Wang, 2005; Choi et al., 2008). Another group of phospholipids, which could potentially interact with NO, are sphingolipids (Guillas et al., 2013). The role of sphingolipids in relation to NO-action on guard cells needs to be probed in detail.

Among the phospholipids, the effect of PA appears to be quite interesting. In plant tissues, PA generated by either PLC or PLD, can inactivate K^+^_in_ channels and promote stomatal closure (Jacob et al., 1999; Uraji et al., 2012). The increase in the levels of PA in *V. faba* guard cells on exposure to NO and prevention of stomatal closure by inhibitors of either PLC or PLD suggested that NO might be involved in the production of PA and stomatal closure (Distéfano et al., 2008). Among the 12 PLD genes of Arabidopsis, PLDα and PLDδ were shown to be involved in stomatal regulation (Zhang et al., 2009b; Distéfano et al., 2012; Uraji et al., 2012). Further description is in the section on “Signaling components in guard cells during NO action.”

## Polyamines

Polyamines are ubiquitous, low molecular weight nitrogenous aliphatic compounds, which regulate several physiological and developmental functions (Kusano et al., 2008). Although the exact mechanisms are not completely understood, polyamines seem to help in plant adaptation to both biotic and abiotic stress (Alcázar et al., 2010). There are indications that polyamines interact with ABA (Alcázar et al., 2006, 2010). The limited reports on the increase in NO production by polyamines are ambiguous. Flores et al. (2008) observed that upregulation of arginase activity reduced the release of NO in *A. thaliana* mutants. In contrast, polyamines elevated NO production in tobacco BY-2 cells and *Ocotea catharinensis* somatic embryo cultures (Santa-Catarina et al., 2007). Among the three polyamines tested, spermine was the most effective in inducing NO production, followed by spermidine and putrescine. Arginine, despite being a precursor molecule for the polyamine biosynthesis, could not increase NO (Tun et al., 2006).

The increase in NO of guard cells by polyamines may be related to H_2_O_2_. Oxidation of putrescine by DAO can facilitate ABA-induced H_2_O_2_ production (An et al., 2008). When polyamines are catabolized by DAO or PAO, H_2_O_2_ is produced as one of the products (Alcázar et al., 2010). Though speculative, it appears reasonable to expect that the polyamine catabolic byproduct of H_2_O_2_ can elevate NO, as NO acts downstream of relation to H_2_O_2_ during stomatal closure (Srivastava et al., 2009). Further studies are required to clarify if polyamines have a direct or indirect effect on the production of NO and ROS in stomatal guard cells.

## Sources of NO

The levels of NO within the cell, depends on the balance between production and scavenging. There is considerable work on the sources of NO in plant tissues, but very little information is available on the modes of scavenging NO. The possible sources of NO production can be categorized as enzymatic or non-enzymatic. Gupta et al. (2011a) summarized the literature on the sources of NO in plants, proposing that seven possible routes of NO production can be identified. In plants, the NR mediated NO production is accepted widely, while there is ambiguity about the role of a true NOS. Neill et al. (2008) reported that ABA-induced NO synthesis in guard cells could be driven by both NOS-like enzyme and NR activity. Nitrate can be reduced to nitrite and then to NO by NR, using NADP(H) as an electron source (Besson-Bard et al., 2008; Baudouin, 2011). However, the capability of NR in NO production is calculated to be only about 1% of its nitrate reduction capacity (Planchet et al., 2005). The root specific Ni-NOR found in purified plasma membranes of tobacco (*Nicotiana tabacum*) roots, has been proposed to be involved in the reduction of apoplastic nitrite to NO (Stöhr and Stremlau, 2006). The role of such plasma membrane bound nitrite: NO reductase (Ni-NOR) in guard cell NO production is yet to be critically assessed.

The NOS-induced NO production is well documented in animal systems, with reports of three isoforms: inducible, neuronal and endothelial NOS (Alderton et al., 2001). However, the existence of true NOS in plants is strongly questioned, because of two major reasons: (i) apparent absence of NOS in the genome of plants, including Arabidopsis; (ii) no convincing evidence for a protein, with NOS-like activity in higher plants. Although proteins with supposedly NOS activity are occasionally reported (Fröhlich and Durner, 2011), their exact identity is questionable. One of the NOS-like enzymes, described earlier (Moreau et al., 2010), turned out to be a GTPase and renamed as NOA. The role of NOA in NO production appears to be a possibility. Despite intense efforts, a true NOS is yet to be discovered in higher plants. The nearest finding is the report on arginine-dependent NOS-like activity in a green alga, *Ostreococcus tauri* (Foresi et al., 2010). The ambiguity on the source of NO extends to SA-mediated NO-production, with reports implicating the importance of NOS-like enzyme (Xin et al., 2003; Sun et al., 2010) or NR (Zottini et al., 2007; Hao et al., 2010). Immediate attention is required to identify the precise enzymatic source of NO production in guard cells, and such information would be applicable to other plant tissues.

There is an additional possibility of NO production by non-enzymatic reactions. Two such instances are: (i) Reduction of nitrite to NO occurred under the acidic and highly reduced conditions, and such NO formation was not impaired by typical NOS inhibitors (Zweier et al., 1999); and (ii) *Rapid* production of NO from nitrite in the incubation medium, *Hordeum vulgare* (barley) aleurone layers further promoted by phenolic compounds (Bethke et al., 2004). However, the relevance of these non-enzymatic NO sources in guard cells are unclear, and these may not be as crucial as enzymatic ones.

Our current knowledge of biological scavenging mechanisms of NO in plants, is quite meagre. Being diffusible, NO can react with several molecules within the cell. Such decrease in NO, due to its highly reactive nature should be considered important. There are reports that GSH and plant hemoglobins, could scavenge NO (Perazzolli et al., 2004; Basu et al., 2010), but the exact enzymatic steps of NO conversion need to be elucidated. The nitrosylation of cellular proteins could be involved in the NO action as well as the maintenance of NO levels. For example, nitrosylation has been found to affect the activity of proteins, such as GAPDH (Lindermayr et al., 2005; Vescovi et al., 2013; Zaffagnini et al., 2013) and outward K^+^-rectifying channels (Sokolovski and Blatt, 2004).

## Signaling components in guard cells during no action

Several signaling components have been identified to act either upstream or downstream of NO. The role of different components was established by usually three sets of evidence: (i) Employing inhibitors or scavengers, (ii) Monitoring the components by suitable fluorescent dyes; and finally (iii) Validation by using mutants deficient in a given component of signal transduction chain (Table 2). The inhibitors related to NO are: cPTIO (scavenger of NO), L-NAME (inhibitor of NOS) and tungstate (inhibitor of NR). In some studies, artificial NO donors such as SNP and GSNO are also used. Studies on real-time monitoring of NO production, during stomatal closure have demonstrated that pH and ROS of guard cells rise before that of NO and stomatal closure occurs subsequently. Such early rise in pH and ROS was observed during stomatal closure induced by ABA, MJ as well as chitosan (Suhita et al., 2004; Gonugunta et al., 2008, 2009; Srivastava et al., 2009). Studies using NO scavenger (cPTIO) or L-NAME and tungstate, inhibitors of “NOS-like” and NR prevented the NO production but not ROS during stomatal closure in epidermal strips. Among the signaling components: PYR/PYL/RCAR (ABA-receptor proteins), ABI1/2 (that help binding to receptor proteins), ROS (generated by NADPH oxidase), pH, G-proteins and PA/PLC/PLDα1 act upstream of NO rise (Sirichandra et al., 2009; Zhang et al., 2009b; Cutler et al., 2010). In contrast to the role of PLDα1, PLDδ is reported at either upstream or downstream of NO production in guard cells (Distéfano et al., 2012; Uraji et al., 2012). Similarly, Ca^2+^ may act at both levels upstream and downstream of NO (Garcia-Mata et al., 2003; Gonugunta et al., 2008).

Unlike other reports, an intriguing observation was that ABI1 and ABI2 might act downstream of the NO in stomatal signaling by ABA in *A. thaliana* guard cells (Desikan et al., 2002). Studies with mutants deficient in ROS production (like *rbohD/F*) and by inhibitors like DPI, confirmed the strong association between ROS and NO (Bright et al., 2006; Neill et al., 2008; Srivastava et al., 2009). The stomatal closure induced by ABA or H_2_O_2_ and associated NO production were impaired in *nia1,nia2* double mutant (Bright et al., 2006). The NO production by microbial elicitors (boehmerin, harpin and INF1) was impaired in *NbrbohA* and *NbrbohB* single and double silenced plants confirming that ROS acted upstream of NO production (Zhang et al., 2009a). Similarly, limited stomatal closure and NO production in response to microbial elicitors (harpin, Nep1, boehmerin) in G-protein (*G*α-, *G*β1-, and *G*β2-) silenced plants of *N. benthamiana* prove that G-proteins facilitate NO production, before stomatal closure (Li et al., 2009a; Zhang et al., 2012b).

The ability of PA to interact with ABI1 and NADPH oxidase (Zhang et al., 2004) implies that PA may act either upstream or downstream of NO. Distéfano et al. (2008, 2010) have established that the rise in NO causes elevation of PA which acts downstream of the NO during stomatal closure in *V. faba*. In the signaling scheme, proposed by Distéfano et al. (2010), ABA-induced NO activates PLC and/or PLD pathways to generate PA (Zhang et al., 2009b; Uraji et al., 2012). One of the products of PLC, namely IP_3_ can induce the release of Ca^2+^ from internal stores leading to stomatal closure. Attention needs to be drawn to reported participation of the PI3 and PI4 kinases (Kolla and Raghavendra, 2007) in bicarbonate-induced NO production. Such pathway is extremely interesting and may represent ROS-independent route of NO-production.

**Figure 1:**
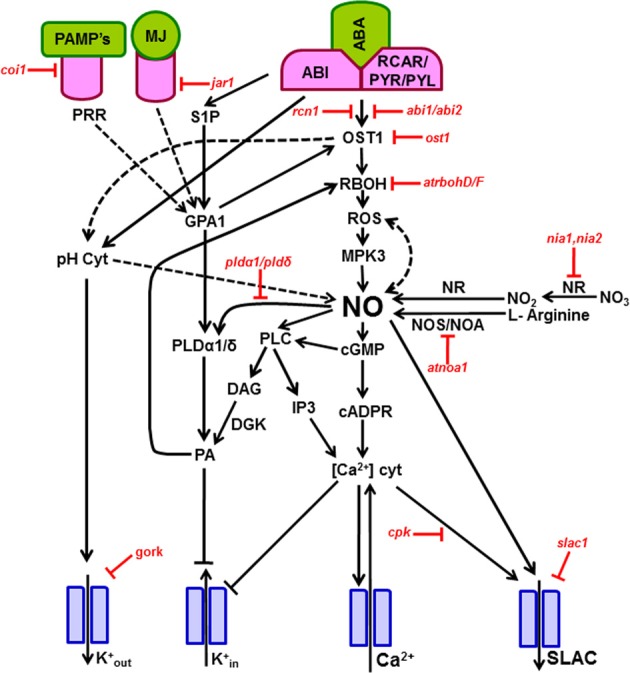
**Signal transduction mechanism involved during stomatal closure induced by ABA, MJ, and microbial elicitors.** The components/secondary messengers induced by either ABA or MJ or elicitors leading to the production of nitric oxide are indicated by forward arrows. The ion channels are represented by blue color. During stomatal signaling mechanism the guard cells upon perception of ABA, MJ, or elicitors, activate NADPH oxidase, leading to a burst of ROS, which leads to a NO burst. The elevation of NO raises the cytosolic free Ca^2+^, through up-regulation of cADPR and cGMP. In turn, the high cytosolic Ca^2+^ causes a down-regulation of K^+^ inward channels and activation of outward anion channels, all leading to stomatal closure. Parallely, NO can increase the levels of PA via modulation of PLD and PLC. Several of these steps are validated by the use of mutants of *Arabidopsis* (indicated by red color), deficient in a particular signaling component. In the mutants, the relevant steps are blocked. The Arabidopsis mutants represented in this Figure are: *abi1/abi2*, ABA-insensitive (ABI1 and ABI2 protein phosphatases); *atrbohD/F*, *A. thaliana* NADPH oxidase catalytic subunit D/F; *atnoa*, *A. thaliana* nitric oxide-associated 1; *coi1*, coronatine-insensitive 1 mutant; *cpk*, calcium-dependent protein kinase; *gork*, guard cell outward rectifying K^+^ channel; *jar1*, JA response 1 mutant; *nia1, nia2*, Nitrate reductase double mutant; *ost1*, open stomata 1 kinase; *pldα1/pldδ*, phospholipase α1/phospholipase δ double mutant; *rcn1*, protein phosphatase 2A regulatory A subunit 1; *slac1*, slow anion channel-associated 1 mutant. A description of these components is given in the section on “Signaling components in guard cells during NO action.” Further information can be seen in Tables 1, 2. Abbreviations are listed in first page. The events demonstrated by experimental evidence are represented by solid arrows. The possible interactions/effects are indicated by broken arrows.

A direct well-known effect of NO is it's up-regulation of Ca^2+^ ion channel activity, promoting the release of Ca^2+^ from intracellular Ca^2+^ stores. Such rise in Ca^2+^ by NO was blocked by antagonists of guanylate cyclase and cADPR indicating that the downstream action of NO is mediated by both cADPR and cGMP. Parallely, the rise in cytosolic free Ca^2+^ inactivates K^+^_in_ channels (blocking K^+^_in_ currents) and activates Cl^−^ ion channels (increasing anion currents), and both events lead to stomatal closure (Garcia-Mata et al., 2003; Sokolovski and Blatt, 2004; Sokolovski et al., 2005). A possible scheme of the signal transduction mechanism involving various components is presented in Figure 1.

Besides their key roles during the rise in NO and subsequent effects, several signaling components tend to interact (Table 3). The best and well known interactions of NO are with ROS, Ca^2+^ and PA, and to some extent, with pH. For e.g., Ca^2+^ stimulates NO production and NO in turn can rise Ca^2+^ levels (Garcia-Mata and Lamattina, 2007). Such dual role of Ca^2+^ is extremely interesting and warrants detailed examination. Similarly, the production of NO and PA promote the levels of each other (Zhang et al., 2009b). There may be a feedback regulation by NO of cytosolic pH, since the rise in NO by SNP increased also the pH of guard cells (Gonugunta et al., 2008, 2009), but there is no convincing evidence of such regulation of guard cell pH by NO during stomatal closure.

**Table 3:** **Interactions of signaling components with NO during modulation of stomatal closure induced by different effectors**.

**Signaling component**	**Type of interaction**	**Plant**	**Effector**	**References**
Cytosolic pH	Precedes NO production	*Pisum sativum*	ABA, MJ and Chitosan	Gonugunta et al., 2008, 2009
		*Arabidopsis thaliana*	Ethylene	Jing et al., 2010
H_2_O_2_	Promotes NO production	*P. sativum*	Chitosan	Srivastava et al., 2009
		*A. thaliana*	ABA	Bright et al., 2006
Ca^2+^	Increases NO production	*Vicia faba*	ABA	Garcia-Mata and Lamattina, 2007
PLDα1	Increases NO production	*A. thaliana*	ABA	Zhang et al., 2009b
PLDδ	Acts downstream of NO	*A. thaliana*	ABA and NO	Distéfano et al., 2012
H_2_S	Depletes NO levels in guard cells	*A. thaliana*	H_2_S	Lisjak et al., 2010
	Functions downstream of NO	*V. faba*	Ethylene	Jing et al., 2012
ABA	NO increases the sensitivity to ABA	*A. thaliana*	NR and NOA	Lozano-Juste and León, 2010
MJ	Elevates endogenous ABA	*A. thaliana*	Methyl jasmonate	Ye et al., 2013

The marked interactions between signaling components, involving NO, constitute a dynamic and complex regulatory network. Because of the complicated nature of signaling network and strong interactions among them, only a few attempts have been made to model these events. Li et al. (2006) presented a dynamic model of signaling components in which NO is produced by NR and NOS-like enzyme, in response to ABA, and the Ca^2+^ mobilized from intracellular sources, could induce stomatal closure. Similarly, Beguerisse-Díaz et al. (2012) proposed a model of interactions between NO and ethylene. These models need to be validated by experimental evidences.

## Concluding remarks

The available literature amply demonstrates that NO is a common signaling component and a converging step for events initiated by ABA, MJ, or elicitors. The upstream components of NO, which rise during ABA action, are broadly understood. For example, ABA binds to PYR/PYL/RCAR proteins and then to PP2C forming a trimeric complex. Due to the non-availability of PP2C, protein kinases are activated to trigger several downstream elements (Cutler et al., 2010; Raghavendra et al., 2010). However, the mechanism of reception and transduction of elicitor signals, particularly the elicitor-receptor interactions, and events leading to NO rise, are not clear and need detailed examination. The levels of NO in guard cells during stomatal closure are usually monitored by using suitable fluorescent dyes, such as DAF-2DA. But these measurements are being debated, since the specificity of fluorescent dyes has been questioned, due to their proneness to artifacts. Efforts are on to reassess and reconcile measurements of NO in plant tissues (Mur et al., 2011). The exact source of NO in plant tissues continues to be a controversial topic. Several possibilities have been identified, such as NR, NIR, NOS-like and even NOA, but the available literature is not convincing enough to assess the relative significance of the different sources (Neill et al., 2008; Gupta et al., 2011a).

A range of highly interesting topics are emerging, studies on which can be quite useful. Among these are: modulation of NO by endogenous plant hormones, such as ABA (Lozano-Juste and León, 2010), role and interaction with other gaseous molecules such as H_2_S and CO, termed gasotransmitters (García-Mata and Lamattina, 2013), and the post-translational modification of downstream proteins by NO or ROS or both (Yoshioka et al., 2011). In summary, further detailed work on the role and source of NO in guard cells promises to be a rewarding exercise and may provide information relevant to other plant tissues.

### Conflict of interest statement

The authors declare that the research was conducted in the absence of any commercial or financial relationships that could be construed as a potential conflict of interest.

## Abbreviations

ABA: abscisic acid
ABI1/2: ABA-insensitive protein phosphfatase 2C type 1/2
cPTIO: 2-phenyl-4,4,5,5-tetramethyl imidazoline-1-oxyl 3-oxide
cADPR: cyclic ADP ribose
CDPK: calcium-dependent protein kinase
CO: carbon monoxide
CO_2_: carbon dioxide
cGMP: cyclic guanosine monophosphate
DAO: diamine oxidase
DGK: diacylglycerol kinase
DAF-2DA: 4,5-diaminofluorescein diacetate
DAG: diacylglycerol
ExtCaM: extra cellular calmodulin
flg22: flagellin 22
GSNO: S-nitrosoglutathione
GAPDH: glyceraldehyde-3-phosphate dehydrogenase
GSH: glutathione
H_2_S: hydrogen sulfide
H_2_O_2_: hydrogen peroxide
MAPK: mitogen-activated protein kinase
MJ: methyl jasmonate
L-NNA: *N*_ω_-nitro-L-arginine
L-NAME: *N*-nitro-L-arginine methyl ester
NR: nitrate reductase
NADPH: Nicotinamide adenine dinucleotide phosphate
NO: nitric oxide
NOS: nitric oxide synthase
NIR: nitrite reductase
NOA: nitric oxide-associated
LPS: lipopolysaccharide
PAO: polyamine oxidase
PAMP: pathogen-associated molecular pattern
PIP_2_: phosphatidylinositol 4,5-bisphosphate
PA: phosphatidic acid
PLD: phospholipase D
PLC: phospholipase C
PP2C: type 2C protein phosphatase
ROS: reactive oxygen species
SA: salicylic acid
SNP: sodium nitroprusside
XOR: xanthine oxidoreductase
YEL: yeast elicitor
PYR/PYL/RCAR: pyrabactin resistance protein1/PYR-like proteins/regulatory components of ABA receptor.
